# Callous-Unemotional Traits Moderate the Relationship Between Irritability and Threatening Responding

**DOI:** 10.3389/fpsyt.2021.617052

**Published:** 2021-11-16

**Authors:** Ru Zhang, Johannah Bashford-Largo, Jennie Lukoff, Jaimie Elowsky, Erin Carollo, Amanda Schwartz, Matthew Dobbertin, Sahil Bajaj, Karina S. Blair, Ellen Leibenluft, R. James R. Blair

**Affiliations:** ^1^Center for Neurobehavioral Research, Boys Town National Research Hospital, Boys Town, NE, United States; ^2^Section on Mood Dysregulation and Neuroscience, Intramural Research Program, National Institute of Mental Health, National Institutes of Health, Bethesda, MD, United States

**Keywords:** threat responsiveness, irritability, callous-unemotional traits, conduct disorder, fMRI

## Abstract

**Background:** Irritability and callous-unemotional (CU; reduced guilt/empathy) traits vary dimensionally in the typically developing population but may be particularly marked in youth with conduct disorder (CD). While these dimensional traits are positively correlated, they have been associated with divergent forms of dysfunction, particularly with respect to threat processing (i.e., irritability with increased, and CU traits with decreased, threat responsiveness). This suggests that interactions between these two dimensions may be complex at the neurobiological level. However, this issue has received minimal empirical attention.

**Methods:** The study included 105 adolescents (typically developing and cases with CD; *N* = 59). They were scanned with fMRI during a looming threat task that involved images of threatening and neutral human faces or animals that appeared to be either looming or receding.

**Results:** Significant *irritability-by-CU traits-by-Direction-by-Emotion interactions* were seen within right thalamus/PAG, left lingual gyrus and right fusiform gyrus; irritability was positively associated with the BOLD response for Looming Threatening vs. Receding Threatening trials, particularly for youth with low CU traits. In contrast, CU traits were *negatively* associated with the same differential BOLD response but particularly for youth showing higher levels of irritability. Similar findings were seen within left ventral anterior and posterior cingulate cortices, though the addition of the interaction with CU traits was only seen at slightly more lenient thresholds.

**Conclusions:** The results support previous work linking irritability to increased, and CU traits to reduced, threat responsiveness. However, for adolescents with high irritability, if CU traits are also high, the underlying neuropathology appears to relate to reduced, rather than increased, threat responsiveness.

## Introduction

According to the fifth edition of Diagnostic and Statistical Manual of Mental Disorders [DSM-5; (1)], the essential feature of conduct disorder (CD) is a repetitive and persistent pattern of behavior in which the basic rights of others, or major age-appropriate social norms, are violated. Youth with conduct problems are at risk of emotional impairment and low social and academic achievement that can have long-term detrimental effects on developmental outcomes (2, 3). Adolescents showing conduct problems are a heterogeneous population who differ in age of onset, form of aggression (reactive or instrumental), comorbidity of affective disorders, treatment response and affective traits (2). Numerous efforts have been made to understand this heterogeneity in an attempt to tailor treatments and improve outcomes. An approach that has gained empirical support in recent years is to focus on affective traits related to CD and their neurobiology.

One area of neurobiological function that received focus is threat processing. Core brain regions implicated in threat processing include those generating the emotional response [amygdala, hypothalamus, periaqueductal gray (PAG), and anterior insula cortex]; those representing the threat (visual and temporal cortices); and those representing emotional valence and maintaining the emotional reaction [ventromedial prefrontal cortex (vmPFC), rostromedial prefrontal cortex (rmPFC), and posterior cingulate cortex (PCC)] (4–9).

Affective traits related to conduct problems include: (i) callous-unemotional (CU) traits (reduced guilt following misbehavior, reduced empathy for others in distress, reduced concern about one's own performance, and shallow or deficient affect) (10); and (ii) irritability—defined as an “increased propensity to exhibit anger relative to one's peers” [(11), p.277] and a “relative dispositional tendency to respond with anger to blocked goal attainment, and includes both mood (trait) and behavioral (reactive state) dysregulation” [(12, 13), p.69; see also (14, 15)]. Work on CU traits and irritability has, for the most part, developed in parallel [though see (4, 15)]. Most studies indicate that the level of CU traits is inversely related to responsiveness to the distress of others and to threat stimuli in the amygdala and related regions such as anterior insula, temporal cortices and vmPFC (16–18). In contrast, neuroscience-based models of irritability typically stress *hyper*activity to threat (4, 11, 19). These findings of threat *hypo*-responsiveness relating to CU traits but threat *hyper*-responsiveness relating to irritability are striking given that CU traits and irritability are *positively* associated [e.g., *r* = 0.463; (20)]. Indeed, irritability may predict later CU levels (21)—though it has also been associated with later depression (22).

The question then becomes how to understand the relationship between the neurobiology and presentation of CU traits vs. irritability. Is irritability underpinned by the same neurobiology whatever the individual's level of CU traits? Or could irritability be underpinned by different neurobiology in individuals with high vs. low CU traits? Given that: (i) higher irritability has been associated with greater threat responsiveness (23–27); but (ii) higher CU traits has been associated with lower threat responsiveness (16–18); and (iii) irritability and CU traits are positively associated, it suggests that the neuro-cognitive models of irritability and/or CU traits may be incomplete for adolescents with high levels of irritability and CU traits. This should manifest as irritability-by-CU traits interactions with respect to BOLD response data such that the relationship between irritability (and/or CU) traits should differ as a function of level of CU traits (and/or irritability).

The current study explores these issues. Specifically, the current study examines the extent to which there is an interaction between level of CU traits and irritability in adolescents, specifically in neural systems responsive to threat. Consistent with RDoC (28, 29), a dimensional approach was taken, with the sample representing the range from typically developing (TD) to a psychiatrically heterogeneous (in terms of co-morbid diagnoses) sample of cases with CD (a psychiatric condition particularly associated with CU traits and irritability). We predicted that regions responsive to threat (e.g., the amygdala, PAG and connected cortical regions; vmPFC and visual and temporal cortices) would show irritability-by-CU traits interactions particularly manifesting at higher levels of irritability and/or CU traits. This prediction was assessed *via* two different analysis approaches. First, a standard task-by-CU traits/irritability ANCOVA analysis of the BOLD responses of the study participants. Second, an interrogation of BOLD response activity within regions implicated in task features (looming threat, threat images), via an independent TD sample, in the study participants (anonymous reviewer's suggestion).

## Methods

### Participants

Study participants included 105 youths aged 14-18 years from a residential care program and the surrounding community (54 from the residential treatment program and 51 from the community): average age = 16.299 (SD = 1.248), average IQ = 99.829 (SD = 9.689), and 63 males. An additional three participants from the residential treatment program were approached but either their parents did not provide consent or they did not assent. Participants were recruited as part of a broader study determining neural correlates of behavioral and emotional problems in youth. Participants were clinically characterized through psychiatric interviews conducted by a licensed, board-certified psychiatrist with the participants and their parents, to adhere closely to common clinical practice. A member of the clinical research team obtained written informed consent and assent. In all cases, youth had the right to decline participation at any time before or during the study. The Boys Town National Research Hospital institutional review board approved this study. For full details on consenting and assenting procedure and the exclusion criterion, see Supplementary Material.

### Measures

#### fMRI Task

**Figure 1:**
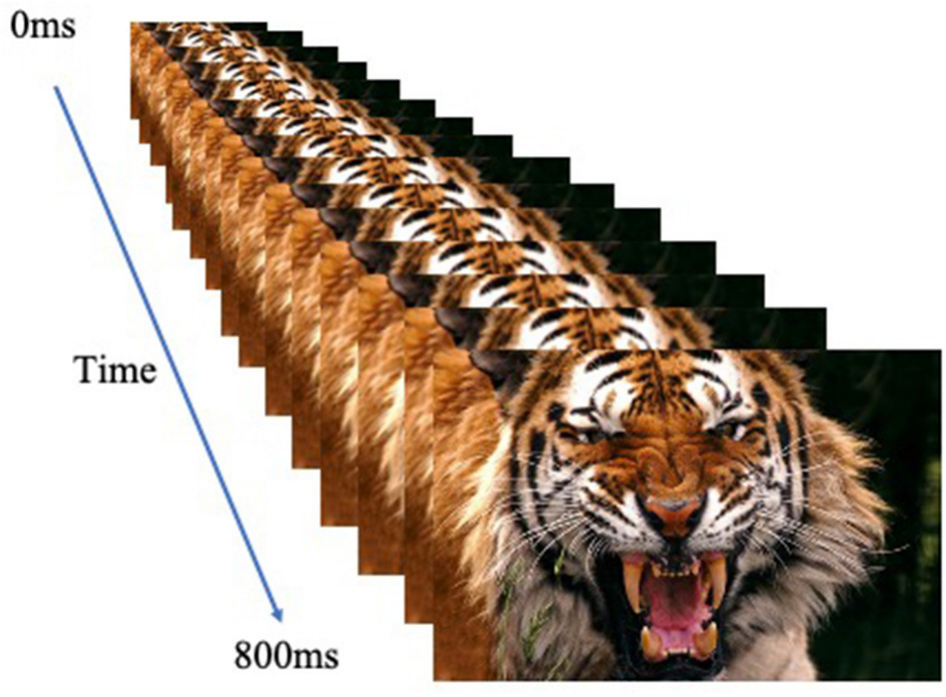
The looming task. Example of the Looming Threatening Animal trial.

The participants performed a looming task [adapted from (30)] and used previously with adolescent participants [e.g., (31–34)]. They were presented with an image that appeared to either loom toward or recede away from them. Images were human or animal faces and were either threatening or neutral. Images were presented rapidly in a series of sixteen 50 ms frames of increasing or decreasing size in the center of the screen to create the effect of either looming (i.e., increasing in size in rapid succession) or receding (i.e., decreasing in size in rapid succession; total stimulus duration: 800 ms); see Figure 1. Stimulus presentations were followed by a fixation point, which was on screen for a jittered duration of 1,250-4,250 ms. The task included 1 block of 160 stimuli (20 of each of the 8 trial types). In order to ensure attention to the task, participants were instructed to press a button with their right index finger as quickly as possible when an image appeared on the screen.

#### Inventory of Callous-Unemotional Traits (ICU)

The ICU is a 24-item self-report scale designed to assess CU traits in youth (35). The construct validity of the ICU has been supported in community and juvenile justice samples (36, 37).

#### Affective Reactivity Index (ARI)

It is a seven-item self-report questionnaire that assesses the youth's irritability during the preceding 6 months (six symptom items and one function impairment item). Prior work has indicated that the ARI is a reliable and valid measure of irritability in youth (14).

Additional measures included the conduct problems subscale from the Strength and Difficulties Questionnaire [SDQ-CP: (38)] and the Reactive Proactive Questionnaire (39)—to provide information on severity of aggression and association of aggression with CU traits and irritability in this population, the Conners ADHD scale (40), to provide information on severity of ADHD symptomatology in this population and the Alcohol Use Disorder Identification Test [AUDIT; (41)] and Cannabis Use Disorder Identification Test [CUDIT; (42)] assessing AUD and CUD symptom levels over the previous 12 months.

### fMRI Parameters

Whole-brain BOLD fMRI data were acquired using a 3.0 Tesla Siemens Skyra scanner. Functional images were taken with a T2^*^ weighted gradient echo planar imaging (EPI) sequence (repetition time = 2,500ms; echo time = 27 ms; 240 mm field of view; 94 × 94 matrix; 90° flip angle). Whole-brain coverage was obtained with 43 axial slices (thickness, 2.5 mm; voxel size 2.6 × 2.6 × 2.5 mm^3^). A high-resolution T1 anatomical scan (MP-RAGE, repetition time = 2,200 ms; echo time = 2.48 ms; 230 mm field of view; 8° flip angle; 256 × 208 matrix; thickness, 1 mm; voxel size 0.9 × 0.9 × 1 mm^3^) in register with the EPI data set was obtained covering the whole brain with 176 axial slices.

### fMRI Analyses

Data were analyzed within the framework of a random effects general linear model (GLM) using Analysis of Functional Neuroimages [AFNI; (43)]. The anatomical scan for each participant was registered to the Talairach and Tournoux atlas (44) and each participant's functional EPI data were registered to their Talairach anatomical scan in AFNI. Functional images were motion corrected to a reference volume and spatially smoothed with a 6-mm full width half maximum Gaussian kernel. The data then underwent time series normalization to a T1 image, and these results were multiplied by 100 for each voxel. Therefore, the resultant regression coefficients are representative of a percentage of signal change from the mean.

Afterward, eight indicator regressors were generated: Receding Neutral Animal, Receding Neutral Human, Receding Threatening Animal, Receding Threatening Human, Looming Neutral Animal, Looming Neutral Human, Looming Threatening Animal, Looming Threatening Human. General Linear Model fitting was performed with the eight regressors listed, six motion regressors, and a regressor modeling a first-order baseline drift function. This produced a β-coefficient and an associated *t*-statistic for each voxel and regressor.

Volumes were censored if there was >0.5 mm motion across adjacent volumes. Participants were excluded due to excessive motion (>20% censored volumes; mean = 0.7%, SD = 1.7%) or low response rate (<65% responses; mean = 91.6%, SD = 6.5%) on the task. There were no significant correlations between ARI scores and censored volumes, average motion per volume, and maximum displacement during scanning within the final sample (*r*'s = 0 to 0.08, *p* > 0.40). However, there was a negative correlation between ICU scores and average motion per volume (*r* = −0.23, *p* = 0.023). The correlations between ICU and censored volumes and maximum displacement were non-significant (*r* = −0.18, −0.01, *p* = 0.082, 0.920).

### Statistical Analyses

#### Clinical Data

Potential relationships between ICU and ARI scores and age, IQ, sex and prescribed medications were examined via zero-order correlation analyses.

#### Behavioral Data

To examine associations between ICU and ARI scores and behavioral performance on the task, we ran a full factorial 2 (Direction: Looming, Receding) by 2 (Type: Animal, Human) by 2 (Emotion: Threatening, Neutral) repeated measures analysis of covariance (ANCOVA) on the response time (RT) data with ICU and ARI scores as continuous covariates. To reduce skewness and kurtosis (and thus disproportionate influence on coefficient estimates of data points in the tails/outliers), a rankit transformation (45) was applied to the ICU and ARI scores. Pre-transformation, these were 1.43 and 1.56 for the ARI and 0.47 and 0.11 for the ICU, respectively. Following transformation these were 0.02 and −0.20 and 0.49 and −0.36, respectively. The rankit transformed ICU and ARI scores were then *z*-scored, and these values were used as continuous covariates in all ANCOVA analyses.

#### BOLD Response Data

1. **Whole brain analysis**
  To examine associations between ICU and ARI scores and dysfunction within brain regions responsive to Direction, stimulus Type or Emotion, we ran a full factorial 2 (Direction: Looming, Receding) by 2 (Type: Animal, Human) by 2 (Emotion: Threatening, Neutral) repeated measures ANCOVA on the BOLD response data with ICU and ARI scores as continuous covariates via 3dMVM within AFNI; i.e., all component main effects, and all two- and three-way interactions were included in the model. Group was not included as a variable in the statistical model as including CD as a covariate in the model would effectively divide the statistical variance between behavioral manifestations (CD behaviors and the socio-affective traits) of this diagnosis. Age, sex and IQ were not included in these models as none of these variables were associated with either ICU or ARI score.
2. **Functional ROI analysis**
  Functional ROIs were derived from the data from an independent sample of 99 TD participants (for details of the sample, see Supplementary Material 3). Specifically, to identify regions involved in threat processing, we conducted a full factorial 2 (Direction: Looming, Receding) by 2 (Type: Animal, Human) by 2 (Emotion: Threatening, Neutral) repeated measures ANOVA on the BOLD response data from this independent sample to identify regions showing a main effect of Direction (Looming > Receding) and/or Emotion (Threatening > Neutral).

BOLD responses within the functional ROIs showing a main effect of Direction (Looming > Receding) and/or Emotion (Threatening > Neutral) to Direction and/or Emotion cues for the study sample was then calculated. Following this, two Region by Direction by Type by Emotion repeated measures ANCOVAs were conducted on these data with ICU and ARI scores as continuous covariates (one for the Direction and one for the Emotion ROIs).

#### Secondary Analyses

Analyses exploring the nature of observed ANOVA and ANCOVA derived interactions were performed within RStudio and freely available online tools. In addition, Steiger's *z*-tests were used to compare the correlations between ICU and ARI scores and RT and BOLD responses to task variables (46).

For irritability-by-CU traits interactions, a bootstrapping procedure (10,000 samples) using the PROCESS macro for SPSS (47) was used to examine how irritability/CU traits moderated the association of trait variables with RT or BOLD response. The Johnson-Neyman technique was used to investigate heterogeneity of the relationships between CU traits and irritability and BOLD responses (48). This technique identified specific ranges of trait scores (e.g., ARI scores) where the relationship between CU level (ICU scores) and RT/BOLD responses was significant (48).

### Secondary Analyses: CD Diagnosis and Potential Confounds

#### CD Diagnosis

To determine the relationship between CD diagnostic status and BOLD response, a full factorial 2 (Group: with CD, TD) by 2 (Direction: Looming, Receding) by 2 (Type: Animal, Human) by 2 (Emotion: Threatening, Neutral) repeated measures ANOVA was conducted on the BOLD response data.

#### Potential Treatment Confounds

Given potential associations of ICU and ARI scores with medication, our ANCOVA analysis was repeated twice, once excluding participants prescribed SSRIs (*N* = 10) and once excluding participants prescribed antipsychotics (*N* = 8).

#### For All BOLD Response Analyses

Correction for multiple comparisons was performed using a spatial clustering operation in AFNI's 3dClustSim utilizing the autocorrelation function (-acf) with 10,000 Monte Carlo simulations for the whole-brain analysis. Spatial autocorrelation was estimated from residuals from the individual-level GLMs. The initial threshold was set at *p* = 0.001. This process yielded an extant threshold of *k* = 23 voxels for the whole brain (multiple comparison corrected *p* < 0.05).

## Results

### Clinical Data

ICU and ARI were positively correlated; see Table 1. While neither were related to age, sex or IQ, both were positively correlated with prescription of antipsychotic and/or SSRI medications (see Table 1). They were also correlated with CUDIT and AUDIT scores—though the strength of correlations (ICU vs. ARI) did not significantly differ (see Table 1).

**Table 1:** Demographic and clinical variables.

	**Mean for CD**	**Mean for TD**	***t* score for TD vs. CD**	***P*-value**	**Mean for the whole sample**	**Std. deviation**	**Range**	**Correlation with ARI**	**Correlation with ICU**	**Steiger's Z**	***P-*value**
Age	16.393	16.177	−0.881	0.381	16.299	1.248	14.07-18.88	0.033	0.085	−0.513	0.608
IQ	98.746	101.217	1.301	0.196	99.829	9.689	79-123	0.074	−0.061	1.332	0.183
ARI	3.627	0.913	−5.798	0.000	2.438	2.728	0-11	–	0.473**	–	–
ICU	26.246	15.767	−6.459	0.000	21.740	9.542	3-51	0.473**	–	–	–
SDQ-CP	7.102	0.087	−27.838	0.000	4.029	3.722	0-10	0.474**	0.486**	−0.140	0.888
RPRS Total	18.614	8.205	−10.901	0.000	13.723	6.778	6-30	0.446**	0.453**	−0.080	0.936
RPRS Reactive	11.114	4.872	−10.691	0.000	8.181	4.097	3-15	0.401**	0.410**	−0.099	0.921
RPRS Proactive	7.500	3.333	−8.892	0.000	5.542	2.977	3-15	0.464**	0.466**	−0.023	0.982
Conners (ADHD)	9.915	0.196	−10.306	0.000	5.657	6.800	0-20	0.531**	0.371**	1.842	0.066
AUDIT	4.89	0.37	−4.306	0.000	2.68	5.392	0-34	0.182	0.274*	−0.841	0.400
CUDIT	10.96	0.49	−7.105	0.000	5.84	8.653	0-29	0.366**	0.479**	−1.137	0.256
	**Percent for CD**	**Percent for TD**			**N**	**Percent**					
Male	62.7%	56.5%	−0.638	0.525	63	60%	–	−0.119	0.018	−1.039	0.299
CD	100%	0%	**–**	–	59	56.2%	–	0.496**	0.546**	−0.450	0.653
ADHD	77.9%	0%	−12.636	0.000	46	43.8%	–	0.508**	0.423**	0.639	0.523
MDD	18.6%	0%	−3.216	0.002	11	10.5%	–	0.495**	0.481**	0.046	0.963
GAD	35.6%	0%	−4.994	0.000	21	20%	–	0.349**	0.405**	−0.256	0.798
No diagnosis	0%	100%	–	–	46	43.8%	–	−0.496**	−0.546**	0.450	0.653
Antipsychotic	13.6%	0%	−2.660	0.009	8	7.6%	–	0.231**	0.268**	−0.084	0.933
Stimulant	23.7%	0%	−3.747	0.000	14	13.3%	–	0.143	0.045	0.319	0.749
SSRI	16.9%	0%	−3.035	0.003	10	9.5%	–	0.091	0.222*	−0.344	0.731

IQ, Intelligent Quotient; ARI, Affective Reactivity Index; ICU, Inventory of Callous-Unemotional Traits; SDQ-CP, Strengths and Difficulties Questionnaire-Conduct Problem; RPRS, Reactive/Proactive Rating Scale; AUDIT, Alcohol Use Disorders Identification Test; CUDIT, Cannabis Use Disorder Test; CD, Conduct Disorder; ADHD, Attention Deficit Hyperactivity Disorder; MDD, Major Depressive Disorder; GAD, Generalized Anxiety Disorder; p = two-tailed significance level of the Steiger's Z calculation (i.e., whether there were significant differences in correlation strength between the ARI scores and ICU scores). *p < 0.05. **p < 0.01.

### fMRI Data

#### Whole Brain Analysis

The covariate-based analysis revealed regions showing the following two interactions (also see Table 2): irritability-by-CU traits-by-Direction-by-Emotion interaction and irritability-by-Direction-by-Emotion interaction. Regions showing main effects of Direction, Type, and Emotion and interactions between these variables are reported in Supplementary Table 1.

**Table 2:** Brain regions displaying significant task variable interactions with ARI and/or ICU, obtained from the irritability-by-CU traits-by-Direction-by-Type-by-Emotion repeated measures ANCOVA.

**Region^**a**^**	**BA**	**Voxels**	**X**	**Y**	**Z^**b**^**	***F*-value**	** $\eta_{\text{p}}^{2}$ **
**Irritability-by-CU traits-by-Direction-by-Emotion**							
R thalamus/PAG	–	37	11	−37	5	34.12	0.262
L lingual gyrus	18	33	−22	−55	2	22.47	0.190
R fusiform gyrus	36	24	38	−34	−25	27.95	0.225
L ACC***	–	208	−13	41	8	18.08	0.159
L PCC***	31	40	−10	−52	26	16.19	0.144
**Irritability-by-Direction-by-Emotion**							
L ACC	24	26	−4	35	5	18.36	0.161
L PCC	–	21	−13	−46	23	27.53	0.223
R amygdala/parahippocampal gyrus***	–	50	20	−16	−19	26.70	0.218

a *According to the Talairach Daemon Atlas (http://www.nitrc.org/projects/tal-daemon/)*,

b Based on the Tournoux and Talairach standard brain template. All results presented at p < 0.001 (corrected p < 0.05) except ^***^(p < 0.005). $\eta_{p}^{2}$ = partial eta squared.

**Figure 2:**
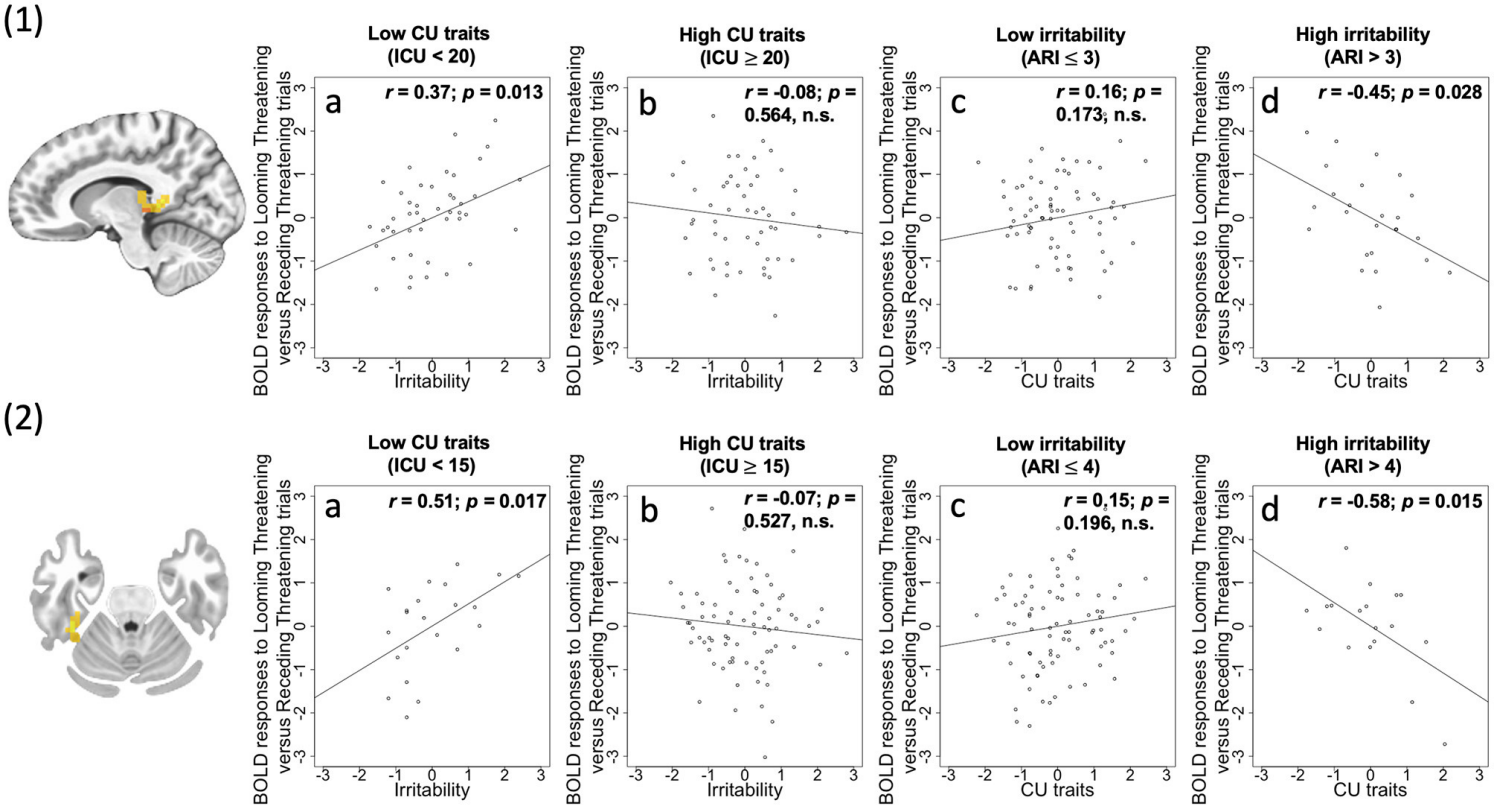
(1) Thalamus/PAG (coordinates: 11, −37, 5) showing a significant irritability-by-CU traits-by-Direction-by-Emotion interaction; (2) Fusiform (coordinates: 38, −34, −25) showing a significant irritability-by-CU traits-by-Direction-by-Emotion interaction. Scatterplots depict the partial correlations within these regions: Adjusted residuals for the BLOM transformed z-scored ARI scores or ICU scores (x-axis) are plotted against adjusted residuals for the average differential BOLD responses to Looming Threatening vs. Receding Threatening trials (y-axis). *r*s are all partial. 1a and 2a: ARI score was significantly positively associated with the Looming Threatening vs. Receding Threatening differential response for participants with lower ICU scores; 1b and 2b: This association was not significant for participants with higher ICU scores; 1c and 2c: ICU score was not significantly correlated with the Looming Threatening vs. Receding Threatening differential response for participants with lower ARI scores; 1d and 2d: This association was significant for participants with higher ARI scores.

*Irritability-by-CU traits-by-Direction-by-Emotion interaction* was significant in three regions: right PAG/thalamus [*F*_(1, 96)_ = 34.12; *p* < 0.001; $\eta_{p}^{2}$ = 0.262], left lingual gyrus [BA 18, *F*_(1, 96)_ = 22.47; *p* < 0.001; $\eta_{p}^{2}$ = 0.190], and right fusiform gyrus [BA 36, *F*_(1, 96)_ = 27.95; *p* < 0.001; $\eta_{p}^{2}$ = 0.225]. Across all the three regions, via the PROCESS macro, we determined that a core component of this interaction reflected a differential response to Looming Threatening trials as opposed to Receding Threatening trials. Irritability was significantly *positively* associated with differential BOLD response to Looming Threatening vs. Receding Threatening trials but at relatively low levels of CU traits (ICU <20, 20, 15; partial *r* = 0.37, 0.27, 0.51; *p* = 0.013, 0.068, 0.017 for the three regions, respectively). In contrast, CU traits were significantly *negatively* associated with differential BOLD response to Looming Threat vs. Receding Threat trials for higher ARI scores (ARI > 3, 4; partial *r* = −0.45, −0.58; *p* = 0.028, 0.015) for right thalamus/PAG and right fusiform gyrus (Figure 2).

**Figure 3:**
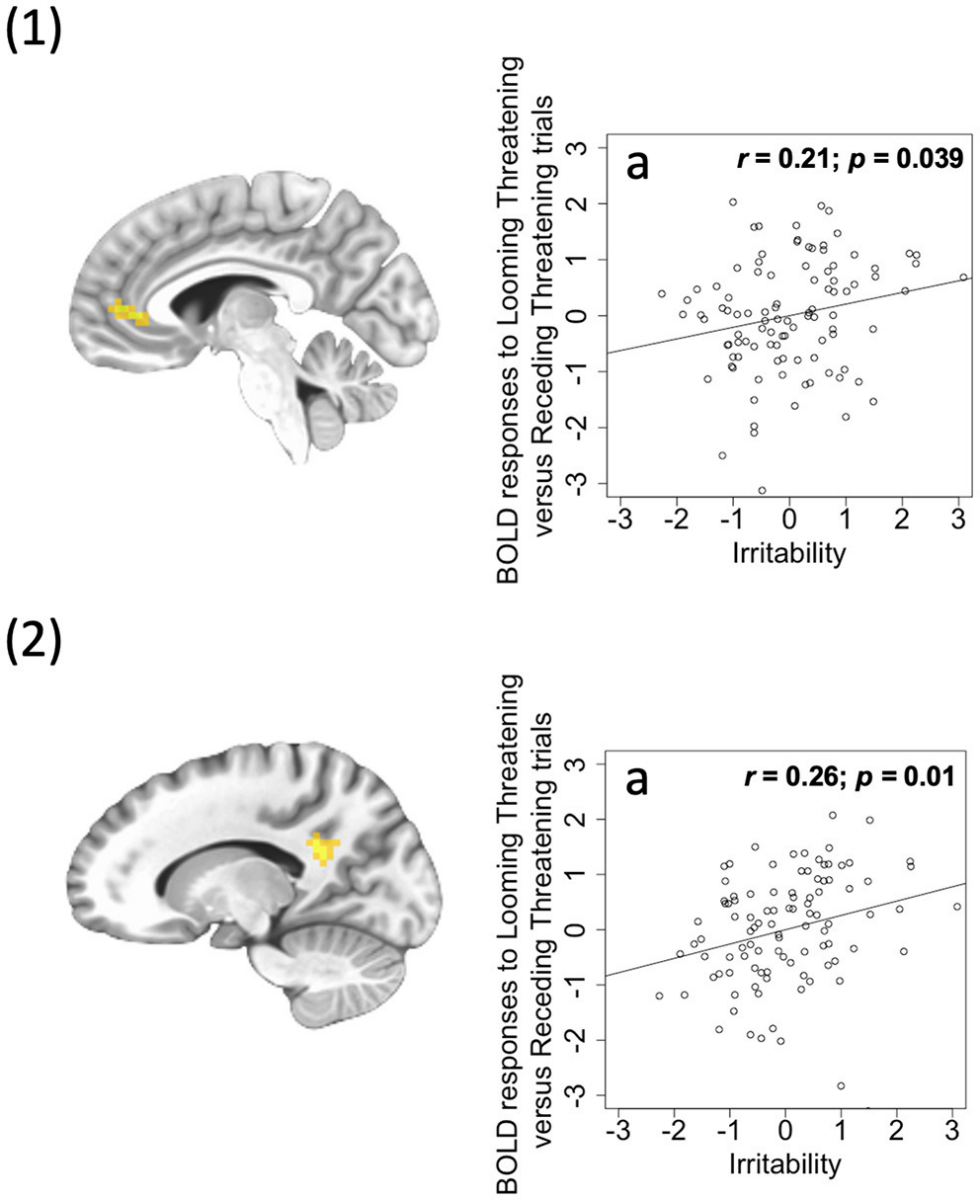
(1) Ventral ACC (coordinates: 26, −4, 35) showing a significant irritability-by-Direction-by-Emotion interaction; (2) PCC (coordinates: 21, −13, −46) showing a significant irritability-by-Direction-by-Emotion interaction. Scatterplots depict the partial correlations within these regions: Adjusted residuals for the BLOM transformed z-scored ARI scores (x-axis) are plotted against adjusted residuals for the average differential BOLD responses to Looming Threatening vs. Receding Threatening trials (y-axis). *r*s are all partial. 1a and 2a: ARI score was significantly positively associated with the Looming Threatening vs. Receding Threatening differential response.

A significant *irritability-by-Direction-by-Emotion interaction* in left ventral anterior cingulate cortex [ACC; BA 24, *F*_(1, 96)_ = 18.36; *p* < 0.001; $\eta_{p}^{2}$ = 0.161] and left PCC [*F*_(1, 96)_ = 27.53; *p* < 0.001; $\eta_{p}^{2}$ = 0.223] was obtained. Within these two regions, irritability was positively correlated with differential BOLD responses to Looming Threats relative to Receding Threats (partial *r* = 0.21, 0.26; *p* = 0.039, 0.01; Figure 3) and Looming Neutral stimuli (partial *r* = 0.29, 0.28; *p* = 0.003, 0.004). It should be noted that both these regions showed *irritability-by-CU traits-by-Direction-by-Emotion interactions* albeit at a slightly more liberal initial threshold (*p* < 0.005; see Supplementary Figure 1). Again, the PROCESS macro revealed that irritability was significantly *positively* associated with differential BOLD response to Looming Threatening vs. Receding Threatening trials but at relatively low CU traits (ICU <20, 22; partial *r* = 0.39, 0.38; *p* = 0.007, 0.004). In contrast, CU traits were again significantly *negatively* associated with differential BOLD response to Looming Threatening vs. Receding Threatening trials particularly for higher ARI scores (ARI > 7, 1; partial *r* = −0.76, −0.34; *p* = 0.048, 0.013; see Supplementary Figure 1).

#### Functional ROI Analysis: BOLD Responses to Task Parameters in an Independent Sample of TD Participants

The ANOVA revealed regions showing main effects of Direction, Type and Emotion. Consistent with previous studies, regions showing increased responses to Looming vs. Receding and Threatening vs. Neutral images included bilateral thalamus, bilateral visual and temporal cortices, bilateral amygdala and vmPFC (though not PCC); see Supplementary Table 2.

#### Association of CU Traits and Irritability in the Test Sample With Activity Within These Functional ROIs

The ANCOVAs for both the Direction (Looming > Receding) and Emotion (Threatening > Neutral) regions revealed significant *irritability-by-CU traits-by-Direction-by-Emotion interactions* [F_(1, 96)_ =8.23 and 5,43; *p* = 0.005 and 0.022; $\eta_{p}^{2}$ = 0.08 and 0.05, respectively). The Process macro revealed that across the regions showing a main effect of Direction, irritability was significantly *positively* associated with differential BOLD response to Looming Threatening vs. Looming Neutral trials but only at lower levels of CU traits (ICU <21 and 20; partial *r* = 0.33 and 0.31; *p* = 0.018 and *p* = 0.028, respectively).

### Behavioral Data

The ANCOVA conducted on the RT data revealed a main effect of Direction [*F*_(1, 96)_ = 72.51; *p* < 0.001; η*p*^2^ = 0.430; participants were faster to respond to Receding than Looming stimuli [386 vs. 419 ms]) and CU traits [*F*_(1, 96)_ = 6.54; *p* = 0.012; η*p*^2^ = 0.064; CU traits were positively associated with RTs (*r* = 0.22, *p* = 0.026)]. In addition, there was a significant irritability-by-CU traits interaction [*F*_(1, 96)_ = 5.04; *p* = 0.027; η*p*^2^ = 0.050]. The PROCESS macro (49) revealed that the increase in RT as a function of CU traits was greater for individuals with an ARI score >1.

### Secondary fMRI Analyses: CD Diagnosis and Potential Confounds

#### CD Diagnosis

Characteristics of the groups are reported in Table 1. Our Group-based ANOVA revealed: (i) a *Group-by-Direction-by-Type-by-Emotion interaction* within left/right cuneus (BA 18 and BA 19, *F*_(1, 103)_ = 20.68; *p* < 0.001; η*p*^2^ = 0.167, see Supplementary Table 3). Within this region, the CD participants, compared to the TD participants, showed less of an increase in response to Looming Threatening Animals relative to Receding Threatening Animals [*t*_(103)_ = 2.467, *p* = 0.015; M[TD] = 0.185; M[CD] = −0.011]; and (ii) a *Group-by-Type interaction* within right PCC [BA 30, *F*_(1, 103)_ = 19.34; *p* < 0.001; η*p*^2^ = 0.158, see Supplementary Table 3). Within this region, CD participants showed significantly less of a discrimination between Human and Animal stimuli than TD participants (M[TD_Animal_] = 0.109; M[TD_Human_] = 0.032; M[CD_Animal_] = 0.040; M[CD_Human_] = 0.049).

#### Potential Treatment Confounds

Given the significant associations of ICU and ARI scores with prescription of antipsychotic and/or SSRI medications (see Table 1), our ANCOVA analysis was repeated twice, once excluding participants prescribed SSRIs (*N* = 10) and once excluding participants prescribed antipsychotics (*N* = 8). These analyses largely mirrored the results of our main analysis (for full details, see Supplementary Tables 4, 5). For completion the analysis was conducted a third time excluding participants prescribed stimulants (*N* = 14). Again this analysis largely mirrored the results of our main analysis (see Supplementary Table 6).

#### The Confound of Potential Suppressor Effects

To ensure that the effects reported above could not be attributed to suppressor effects, we re-ran our main analysis twice; once with only Rankit-transformed ARI score as a covariate, a second time with only Rankit-transformed ICU score as a covariate. The ARI ANCOVA largely mirrored the regions showing irritability-by-Direction-by-Emotion interactions (see Supplementary Table 7). The ICU ANCOVA showed CU traits-by-Direction-by-Type interactions (see Supplementary Table 8).

## Discussion

In this study, we investigated the extent to which there is an interactive association between level of CU traits and irritability in adolescents with respect to neural systems responsive to threat in a sample representing the range from typically developing to cases with CD (i.e., a psychiatric condition particularly associated with CU traits and irritability). There were two main findings: (1) Across right thalamus/PAG, left lingual gyrus and right fusiform gyrus, irritability was significantly positively associated with differential BOLD response to Looming Threatening vs. Receding Threatening trials but at relatively low CU traits. In contrast, CU traits were significantly negatively associated with differential BOLD response to Looming Threatening vs. Receding Threatening trials particularly for individuals with higher ARI scores for right thalamus/PAG and right fusiform gyrus; and (2) Irritability was significantly positively associated with responding within left ventral ACC and left PCC to Looming Threats relative to Receding Threats (though, at slightly more lenient initial thresholds, both regions were showing the same interactions of irritability and CU traits as right thalamus/PAG, left lingual gyrus and right fusiform gyrus).

We predicted that irritability would be related to heightened threat responsiveness within regions involved in responding to threat (the amygdala, PAG and connected cortical regions). Previous work has reported heightened responsiveness within these regions to emotional stimuli in participants with elevated irritability (23–27). In the current study, irritability (at least in those with lower CU traits) was associated with increased responses to approaching threats (relative to comparison conditions) within the amygdala and PAG, fusiform and lingual gyrus and ventral ACC and PCC (albeit with respect to the amygdala at a slightly more liberal threshold). In short, irritability was associated with increased responses within regions included in the acute threat response [amygdala and PAG; (4, 50)], regions representing visual stimuli that are recipients of priming from the amygdala to emotional stimuli [lingual and fusiform cortices; (26, 27)] and regions representing the value of emotional stimuli [and perhaps organizing regulatory responding; vACC and PCC; (51–54)]. These findings are consistent with a view that a core component of irritability is a dysregulated acute threat response such that the individual is more likely to express reactive aggression to a provocation than freezing or avoidance (5, 50, 55).

However, it should be noted that for these regions, the association between irritability and threat responsiveness was moderated by level of CU traits (though for some at slightly more liberal thresholds). Specifically, the positive relationship between ARI score and threat responsiveness was more marked for those with lower CU traits. In most respects, this was anticipated. ARI score and CU traits have been relatively consistently related to inverse forms of psychopathology; increased vs. decreased threat responsiveness, respectively (4, 15). Yet, both are associated with similar symptoms – specifically, anger and reactive aggression (4, 15, 56). It is argued that irritability and reactive aggression can result from increased threat responsiveness; the individual responds with rage rather than freeze or flight to provocation (5, 50, 55). But it is also argued that reactive aggression and anger can result from dysfunctional modulation of threat response circuitry and impaired decision-making regarding the value of future response options; the individual fails to represent the “badness” of the action and so is more likely to commit the non-optimal act (4). We assume that for participants showing irritability, but with low CU traits, their level of irritability reflected the existent hyper-threat responsive psychopathology. However, for many of the participants with higher levels of irritability, the pathology underling their behavior might reflect either hyper- or hypo- threat responsiveness. Indeed, within participants with high ARI scores, the negative association between CU traits and threat responsiveness was most marked. Amongst these individuals, we would argue, the more CU traits the individual showed, the more their reactive aggression and anger was driven by the pathophysiology underpinning CU traits; i.e., dysfunctional representation of the “badness” of anger displays (i.e., the “victims” distress at the anger display and/or the negative consequences following displays of anger for the displayer).

It is worth briefly considering development. Within the sample itself, age plays a relatively minor role (the range is rather narrow 14-18 years; see Supplementary Table 9). Moreover, our main finding, that increased threat responsiveness was associated with irritability unless participants were showing clinically significant levels of CU traits, is unlikely to be highly age-dependent. There are no established theoretical reasons for why this interaction might manifest differently across development. However, heightened threat responsiveness could reflect a failure to mature appropriate emotion regulation mechanisms. Emotion regulation capacities develop substantially across adolescence (57–59). The heightened threat responsiveness might reflect a relative failure in emotion regulation development that can put the individual at increased risk for irritability.

The results for the Group-based ANOVA are worth a brief note. Previous work with aggressive adolescents has mostly focused on distress cues, as opposed to threat responsiveness, in youth with disruptive behavior disorders (DBDs) (60–62). However, there have been studies focusing on responses to threat stimuli in youth with DBDs (16, 63–66). Most of these studies have reported reduced threat responsiveness in adolescents with CD/oppositional defiant disorder [though see (63, 66)]. Our finding of reduced responsiveness in the adolescents with CD relative to comparison adolescents to Looming, Threatening Animals relative to Receding, Threatening Animals within left/right cuneus is consistent with this literature. Importantly, though, this hints that the pathophysiology underpinning the slight majority of cases in this sample was the reduced threat responsiveness putatively underpinning CU traits (as the group of adolescents with CD showed some indication of reduced threat responsiveness). Irritability is seen as a highly cross-diagnostic trait and reported to be increased not only in those showing conduct problems but frequently in cases with mood and anxiety conditions (11, 19, 67). A unique feature of CD is the increased risk for instrumental, goal-directed aggression, something not typically associated with irritability but highly associated with CU traits (4). Of course, as our data reflect, a significant proportion of participants with CD are instead underpinned by the heightened threat responsiveness, the pathophysiology purportedly associated with irritability. As such, it is possible that locations sampling CD populations whose predominant feature is irritability rather than CU traits might report rather different results [cf. (63)].

The current results have clinical implications. Externalizing behaviors are a leading cause of child and adolescent referrals to mental health clinicians (68). Both CU traits and irritability are commonly associated with externalizing behavior (10, 69). The current data suggest a treatment target for irritability if CU traits are low—heightened threat responsiveness. Treatments reducing threat responsiveness might be useful for patients with elevated irritability but not co-presenting CU traits [e.g., (70, 71)]. But, importantly, the current data suggest the situation is more complex if the patient also presents with CU traits at a clinically concerning level. Indeed, the current data suggest that amongst patients with higher irritability, the greater the level of their CU traits, the less hyper-threat responsiveness is a problem. Indeed, amongst these patients, increasing, rather than decreasing emotionality, might be a treatment target [cf. (33)]—though it is unclear currently how this might be achieved.

Four caveats should be considered with respect to the current results. First, there were significant associations between ICU and ARI scores and prescriptions of antipsychotic and/or SSRI medications (see Table 1). As such, the current results might reflect influence of these prescriptions rather than irritability and/or CU traits. Ameliorating this concern is the fact that the secondary analyses that we conducted with participants prescribed either antipsychotic or SSRI medications excluded did not significantly change our results (see Supplementary Tables 4, 5). These data suggest prescribed medications did not significantly confound the current results. Second, one surprising feature in the results was that while CU traits were significantly negatively related to threat responsiveness in participants with higher irritability, they were not in participants with lower irritability. We would have expected that CU traits would have been negatively related to threat responsiveness across the range of irritability scores. Future work will be necessary to determine the robustness of these data and their interpretation. Third, diagnoses followed clinical practice that included an interview by a board-certified psyhchiatrist rather than the implementation of a structured or semi-structured diagnostic interview. While this could raise concern regarding the CD diagnoses, it is important to note: (i) that these diagnoses were supported by the SDQ-CP scores; and (ii) that the main goal of this work was to investigate neural signatures related to CU traits and irritability *across* the sample irrespective of diagnostic status/comorbid conditions. Fourth, there were associations between AUD and CUD symptom severity and both CU traits and irritability. However, it is important to note that the strengths of these associations did not differ (ICU vs. ARI). As such, AUD and CUD symptom severity is less likely to underpin the dissociable CU trait and irritability effects reported here.

In conclusion, irritability was associated with increasing responsiveness in regions that manifest three different functions in this study: (1) thalamus/PAG and amygdala generating response to threat, (2) lingual gyrus and fusiform gyrus representing threat visually, and (3) ACC and PCC maintaining emotional valence and emotional reaction. For most of these regions, this association was moderated by level of CU traits and was most marked for participants with low CU traits. For participants showing higher levels of irritability, CU traits were inversely related to emotional responsiveness for many of these regions. These data indicate that high levels of irritability might be manifested as a consequence of heightened threat responsiveness but also due to the pathophysiology associated with CU traits.

## Data Availability Statement

The original contributions presented in the study are included in the article/Supplementary Material, further inquiries can be directed to the corresponding author/s.

## Ethics Statement

The studies involving human participants were reviewed and approved by the Boys Town National Research Hospital Institutional Review Board. Written informed consent to participate in this study was provided by the participants' legal guardian/next of kin.

## Author Contributions

All authors have contributed in a scientifically substantial way to this manuscript.

## Funding

This research was in part supported by the National Institute of Mental Health under award number K22-MH109558 (JB-L). The funders had no role in the design and conduct of the study; collection, management, analysis, and interpretation of the data; preparation, review, or approval of the manuscript; and decision to submit the manuscript for publication.

## Conflict of Interest

The authors declare that the research was conducted in the absence of any commercial or financial relationships that could be construed as a potential conflict of interest.

## Publisher's Note

All claims expressed in this article are solely those of the authors and do not necessarily represent those of their affiliated organizations, or those of the publisher, the editors and the reviewers. Any product that may be evaluated in this article, or claim that may be made by its manufacturer, is not guaranteed or endorsed by the publisher.
