# Supplementary material for: Callous-Unemotional Traits Moderate the Relationship Between Irritability and Threatening Responding
Source: Front Psychiatry. 2021 Nov 16;12:617052. doi: 10.3389/fpsyt.2021.617052 (PMC8635046; doi:10.3389/fpsyt.2021.617052)
Supplement: Supplementary file 1 [file Data_Sheet_1.docx]

**Supplemental Material**

1. **Consenting and assenting procedures**

The Boys Town National Research Hospital institutional review board approved this study. A doctoral level researcher or a member of the clinical research team obtained written informed consent and assent. In all cases, youth had the right to decline participation at any time before or during the study. With respect to community participants, informed consent was obtained from the youths’ parents/legal guardians at the beginning of the on-site screening. At this time, the consent document was reviewed in detail and the parents/legal guardians had the opportunity to have their questions answered before being asked to sign the consent form. After that, informed assent was obtained from the youths themselves. This procedure differed slightly for youth recruited from the Boys Town campus. Consent was typically obtained from parents during or shortly after the child’s arrival at Boys Town. Assent was obtained from the youth in a separate session, 5-10 days after parental consent had been obtained.

1. **Exclusion criteria**

Exclusion criteria included IQ < 75 assessed with the Wechsler Abbreviated Scale of Intelligence (WASI two-subtest form; Wechsler, 2011), pregnancy, non-psychiatric medical conditions that require the use of medication that may have psychotropic effects (e.g., beta blockers or steroids), current psychosis, pervasive developmental disorders, Tourette’s disorder, neurological disorders, presence of metallic objects in the body (e.g., metal plates, pacemakers, etc.), and claustrophobia. Current psychiatric conditions (other than psychotic disorders or pervasive developmental disorders) were not exclusionary. Use of psychotropic medications for psychiatric indications (e.g., stimulants, selective serotonin reuptake inhibitors) were not exclusory. However, participants on stimulant medication were asked to withhold medication on the morning of the scan.

1. **Independent sample of TD participants**

The data from an independent sample of 99 TD participants (M_age_=12.88, SD= 2.06; M_IQ_=110.01, SD=2.06; 56 males) was analyzed on this task. This revealed regions showing main effects of Direction, Emotion and Type (see Table S2). These regions heavily mirrored those showing main effects of Direction, Emotion and Type in our main analysis (see Table S1).

Table S1. Significant areas of activation obtained from the irritability-by-CU traits-by-Direction-by-Type-by-Emotion repeated measures ANCOVA for all the participants.

| **Region^a^** | **BA** | **Voxels** | **X** | **Y** | **Z^b^** | ***F*-value** | $\boldsymbol{\eta}_{\boldsymbol{p}}^{\boldsymbol{2}}$ |
| --- | --- | --- | --- | --- | --- | --- | --- |
|  |  |  |  |  |  |  |  |
| ***Direction*** |  |  |  |  |  |  |  |
|  |  |  |  |  |  |  |  |
| *Looming > Receding* |  |  |  |  |  |  |  |
| L/R fusiform gyrus/occipital gyrus/cuneus/lingual gyrus | -- | 4376 | 38 | -67 | -10 | 221.16 | 0.697 |
| L thalamus | 27 | 45 | -19 | -28 | -1 | 54.00 | 0.360 |
| R amygdala/parahippocampal gyrus | -- | 26 | 20 | -4 | -13 | 22.74 | 0.192 |
| L/R cerebellar tonsil | -- | 31 | -1 | -52 | -31 | 29.44 | 0.235 |
|  |  |  |  |  |  |  |  |
| *Looming < Receding* |  |  |  |  |  |  |  |
| L dorsomedial prefrontal cortex (dmPFC) | 6 | 82 | -1 | -10 | 59 | 24.92 | 0.206 |
| R anterior insula cortex | 13 | 84 | 47 | 8 | 11 | 32.28 | 0.252 |
| L anterior insula cortex | 13 | 33 | -31 | 14 | 14 | 26.36 | 0.215 |
| L precentral gyrus | 44 | 40 | -52 | 8 | 11 | 28.65 | 0.230 |
| R superior temporal gyrus/ inferior parietal lobule | 42/40 | 865 | 65 | -28 | 17 | 63.35 | 0.398 |
| L superior temporal gyrus/ inferior parietal lobule | 42/40 | 448 | -55 | -34 | 17 | 56.24 | 0.369 |
| R precuneus | 7 | 27 | 8 | -64 | 44 | 27.79 | 0.225 |
| L precuneus | 7 | 23 | -10 | -61 | 44 | 21.42 | 0.182 |
| ***Type*** |  |  |  |  |  |  |  |
|  |  |  |  |  |  |  |  |
| *Human > Animal* |  |  |  |  |  |  |  |
| L/R vmPFC/ACC | 10/32 | 211 | 8 | 41 | -7 | 53.71 | 0.359 |
| L/R cingulate gyrus/precuneus | 31/7 | 245 | -4 | -52 | 32 | 35.51 | 0.270 |
| R superior temporal gyrus | 39 | 203 | 47 | -55 | 20 | 46.56 | 0.327 |
| L middle temporal gyrus | 39 | 67 | -49 | -67 | 29 | 24.48 | 0.203 |
| R middle temporal gyrus | 21 | 28 | 59 | -1 | -10 | 25.47 | 0.210 |
| L middle temporal gyrus | 21 | 24 | -55 | -4 | -10 | 27.34 | 0.222 |
| L amygdala/parahippocampal gyrus | -- | 39 | -16 | -7 | -10 | 52.86 | 0.355 |
|  |  |  |  |  |  |  |  |
| *Human < Animal* |  |  |  |  |  |  |  |
| L precentral gyrus | 9 | 27 | -43 | 5 | 35 | 19.38 | 0.168 |
| L/R parahippocampal gyrus /fusiform gyrus/occipital gyrus/lingual gyrus/cuneus | -- | 7035 | -28 | -31 | -16 | 278.36 | 0.743 |
| R thalamus | -- | 32 | 23 | -25 | 2 | 50.18 | 0.343 |
|  |  |  |  |  |  |  |  |
| ***Emotion*** |  |  |  |  |  |  |  |
| *Threatening > Neutral* |  |  |  |  |  |  |  |
| R middle occipital gyrus/fusiform gyrus | 19/37 | 333 | 35 | -79 | 10 | 49.24 | 0.339 |
| L inferior occipital gyrus | 18 | 264 | -28 | -85 | -7 | 48.01 | 0.333 |
| L fusiform gyrus | 20 | 152 | -37 | -40 | -16 | 44.57 | 0.317 |
|  |  |  |  |  |  |  |  |
| *Threatening < Neutral* |  |  |  |  |  |  |  |
| R middle frontal gyrus | 10 | 49 | 32 | 41 | 17 | 25.28 | 0.208 |
| R superior temporal gyrus | 42 | 55 | 65 | -25 | 11 | 28.30 | 0.228 |
| L superior temporal gyrus | 42 | 23 | -58 | -25 | 14 | 22.92 | 0.193 |
| L cuneus | 18 | 170 | -4 | -73 | 20 | 26.24 | 0.215 |
| R culmen | -- | 32 | 8 | -58 | -7 | 23.71 | 0.198 |
| R lentiform nucleus | -- | 30 | 14 | -7 | 2 | 35.56 | 0.270 |
|  |  |  |  |  |  |  |  |
| ***Direction-by-Type*** |  |  |  |  |  |  |  |
|  |  |  |  |  |  |  |  |
| L lingual gyrus | 18 | 27 | -10 | -76 | -1 | 21.50 | 0.183 |
|  |  |  |  |  |  |  |  |
| ***Direction-by-Emotion*** |  |  |  |  |  |  |  |
| L middle occipital gyrus | 19 | 25 | -34 | -79 | -7 | 22.26 | 0.188 |
|  |  |  |  |  |  |  |  |
| ***Type-by-Emotion*** |  |  |  |  |  |  |  |
|  |  |  |  |  |  |  |  |
| R cuneus | -- | 111 | 5 | -61 | 8 | 21.48 | 0.183 |
| L lingual gyrus/inferior occipital gyrus | 18 | 112 | -25 | -91 | -4 | 33.88 | 0.264 |
| R inferior occipital gyrus/lingual gyrus | 18 | 85 | 26 | -91 | -7 | 27.98 | 0.226 |
| L lingual gyrus | 19 | 55 | -16 | -55 | -1 | 20.03 | 0.173 |
| R middle occipital gyrus | 19 | 39 | 50 | -70 | 8 | 22.25 | 0.188 |
|  |  |  |  |  |  |  |  |

Key to Table S1: ^a^ According to the Talairach Daemon Atlas (<http://www.nitrc.org/projects/tal-daemon/>), ^b^ Based on the Tournoux & Talairach standard brain template. All results presented at *p* < 0.001 (corrected *p* < 0.05). $\eta_{p}^{2}$ = partial eta squared.

Table S2. Significant areas of activation obtained from the Direction-by-Type-by-Emotion repeated measures ANOVA for the independent TD sample.

| **Region^a^** | **BA** | **Voxels** | **X** | **Y** | **Z^b^** | ***F*-value** | $\boldsymbol{\eta}_{\boldsymbol{p}}^{\boldsymbol{2}}$ |
| --- | --- | --- | --- | --- | --- | --- | --- |
|  |  |  |  |  |  |  |  |
| ***Direction*** |  |  |  |  |  |  |  |
|  |  |  |  |  |  |  |  |
| *Looming > Receding* |  |  |  |  |  |  |  |
| 1L/R fusiform gyrus/occipital gyrus/cuneus/lingual gyrus | -- | 3984 | 29 | -43 | -13 | 172.55 | 0.638 |
| L thalamus | -- | 54 | -19 | -22 | -1 | 75.00 | 0.434 |
| R thalamus | -- | 35 | 20 | -25 | 2 | 48.58 | 0.331 |
| R amygdala/parahippocampal gyrus | 28 | 49 | 20 | -4 | -13 | 43.09 | 0.305 |
| R lentiform nucleus | -- | 25 | 20 | 5 | -1 | 26.19 | 0.211 |
| L/R vmPFC*** | 11 | 25 | 2 | 35 | -10 | 16.219 | 0.022 |
|  |  |  |  |  |  |  |  |
| *Looming < Receding* |  |  |  |  |  |  |  |
| R middle frontal gyrus | 9 | 54 | 35 | 29 | 35 | 21.13 | 0.177 |
| R middle temporal gyrus | -- | 69 | 38 | -58 | 5 | 29.25 | 0.230 |
| R inferior parietal lobule | 40 | 77 | 44 | -43 | 50 | 22.58 | 0.187 |
| R precuneus | 7 | 61 | 5 | -61 | 59 | 23.94 | 0.196 |
| R inferior occipital gyrus | 18 | 40 | 26 | -91 | -7 | 48.90 | 0.333 |
| ***Type*** |  |  |  |  |  |  |  |
|  |  |  |  |  |  |  |  |
| *Human > Animal* |  |  |  |  |  |  |  |
| L/R vmPFC | 10 | 164 | 5 | 44 | -7 | 45.26 | 0.316 |
| L/R ACC | 25 | 29 | 5 | 9 | -5 | 42.18 | 0.301 |
| R superior frontal gyrus | 9 | 52 | 5 | 56 | 26 | 25.04 | 0.204 |
| R middle temporal gyrus | -- | 257 | 44 | -43 | 8 | 47.75 | 0.328 |
| R middle temporal gyrus | 21 | 32 | 59 | -7 | -13 | 28.12 | 0.223 |
| R precuneus | 31 | 285 | 2 | -49 | 32 | 31.97 | 0.246 |
| L inferior parietal lobule | 7 | 77 | -43 | -64 | 44 | 21.32 | 0.179 |
|  |  |  |  |  |  |  |  |
| *Human < Animal* |  |  |  |  |  |  |  |
| L/R parahippocampal gyrus /fusiform gyrus/occipital gyrus/lingual gyrus/cuneus | -- | 8080 | -31 | -22 | -19 | 397.14 | 0.802 |
|  |  |  |  |  |  |  |  |
| ***Emotion*** |  |  |  |  |  |  |  |
|  |  |  |  |  |  |  |  |
| *Threatening > Neutral* |  |  |  |  |  |  |  |
| R middle occipital gyrus | 18 | 554 | 29 | -85 | -1 | 86.70 | 0.469 |
| L middle occipital gyrus/fusiform gyrus | 18 | 545 | -34 | -82 | -10 | 72.48 | 0.425 |
| L amygdala/parahippocampal gyrus*** | 34 | 33 | -25 | 2 | -16 | 25.87 | 0.211 |
|  |  |  |  |  |  |  |  |
| *Threatening < Neutral* |  |  |  |  |  |  |  |
| R cuneus | 18 | 91 | 8 | -82 | 17 | 21.82 | 0.182 |
|  |  |  |  |  |  |  |  |
| ***Direction-by-Type*** |  |  |  |  |  |  |  |
|  |  |  |  |  |  |  |  |
| R fusiform gyrus | 19 | 71 | 29 | -52 | -7 | 33.87 | 0.257 |
| L/R lingual gyrus/cuneus | 19 | 506 | -16 | -64 | -4 | 32.69 | 0.250 |
| L middle occipital gyrus | 19 | 82 | -46 | -61 | -4 | 36.77 | 0.273 |
|  |  |  |  |  |  |  |  |
| ***Emotion-by-Type*** |  |  |  |  |  |  |  |
|  |  |  |  |  |  |  |  |
| L cuneus | 17 | 67 | -10 | -76 | 11 | 19.30 | 0.165 |
| R cuneus | 31 | 37 | 14 | -73 | 26 | 21.54 | 0.180 |
| R middle occipital gyrus | 37 | 26 | 50 | -70 | 5 | 24.37 | 0.199 |
| R inferior occipital gyrus | 18 | 256 | 29 | -85 | -7 | 43.98 | 0.310 |
| L lingual gyrus | 17 | 170 | -22 | -91 | -4 | 47.82 | 0.328 |
| R culmen | 18 | 41 | 11 | -64 | -4 | 22.04 | 0.184 |
|  |  |  |  |  |  |  |  |

Key to Table S2: ^a^ According to the Talairach Daemon Atlas (<http://www.nitrc.org/projects/tal-daemon/>), ^b^ Based on the Tournoux & Talairach standard brain template. All results presented at *p* < 0.001 (corrected *p* < 0.05) except *** (*p* < 0.005). $\eta_{p}^{2}$ = partial eta squared.

Table S3. Significant areas of activation obtained from the 2 (Group: CD, TD) by 2 (Direction: Looming, Receding) by 2 (Type: Animal, Human) by 2 (Emotion: Threatening, Neutral) repeated measures ANOVA for all the participants.

| **Region^a^** | **BA** | **Voxels** | **X** | **Y** | **Z^b^** | ***F*-value** | $\boldsymbol{\eta}_{\boldsymbol{p}}^{\boldsymbol{2}}$ |
| --- | --- | --- | --- | --- | --- | --- | --- |
|  |  |  |  |  |  |  |  |
| ***Group-by-Type*** |  |  |  |  |  |  |  |
|  |  |  |  |  |  |  |  |
| R PCC | 30 | 23 | 8 | -61 | 14 | 19.34 | 0.158 |
|  |  |  |  |  |  |  |  |
| ***Group-by-Direction-by-Type-by-Emotion*** |  |  |  |  |  |  |  |
|  |  |  |  |  |  |  |  |
| L/R cuneus | 18/19 | 48 | 2 | -79 | 29 | 20.68 | 0.167 |
|  |  |  |  |  |  |  |  |
| ***Direction*** |  |  |  |  |  |  |  |
|  |  |  |  |  |  |  |  |
| R vmPFC/ACC | 11/10 | 29 | 5 | 41 | -10 | 21.47 | 0.172 |
| L dmPFC | 6 | 100 | -4 | -7 | 56 | 26.84 | 0.207 |
| L inferior frontal gyrus | 9 | 25 | -34 | 8 | 29 | 21.60 | 0.173 |
| R anterior insula cortex | 13 | 61 | 47 | 8 | 11 | 29.68 | 0.224 |
| L insula cortex | 13 | 28 | -31 | 14 | 17 | 25.90 | 0.201 |
| R inferior parietal lobule/superior temporal gyrus | 40/42 | 824 | 56 | -31 | 35 | 63.85 | 0.383 |
| L superior temporal gyrus/ inferior parietal lobule | 42/40 | 415 | -55 | -34 | 17 | 59.40 | 0.366 |
| L/R fusiform gyrus/occipital gyrus/cuneus/lingual gyrus | -- | 5135 | 29 | -34 | -16 | 280.12 | 0.731 |
| R precuneus | 7 | 38 | 8 | -67 | 41 | 23.05 | 0.183 |
| L precuneus | 7 | 26 | 10 | -61 | 41 | 22.31 | 0.178 |
| L/R cerebellar tonsil | -- | 53 | -1 | -49 | -34 | 40.79 | 0.284 |
| R amygdala/parahippocampal gyrus | 28 | 100 | 20 | -4 | -13 | 54.23 | 0.345 |
| L amygdala/parahippocampal gyrus | 28 | 74 | -19 | -4 | -10 | 37.21 | 0.265 |
| ***Type*** |  |  |  |  |  |  |  |
|  |  |  |  |  |  |  |  |
| L/R vmPFC/ACC | 10/32 | 189 | 8 | 41 | -7 | 45.69 | 0.307 |
| L orbitofrontal cortex | 47 | 34 | -22 | 32 | -1 | 27.98 | 0.214 |
| L/R cingulate gyrus | 31 | 261 | 5 | -55 | 26 | 36.16 | 0.260 |
| L inferior frontal gyrus | 9 | 51 | -43 | 5 | 32 | 23.98 | 0.189 |
| L middle frontal gyrus | 46 | 50 | -40 | 32 | 20 | 24.34 | 0.191 |
| R caudate | 25 | 30 | 2 | 8 | -1 | 31.19 | 0.232 |
| R superior temporal gyrus | 39 | 176 | 56 | -61 | 20 | 44.28 | 0.301 |
| L middle temporal gyrus | 39 | 40 | -49 | -67 | 29 | 23.18 | 0.184 |
| R middle temporal gyrus | 21 | 24 | 59 | -1 | -13 | 24.69 | 0.193 |
| L/R parahippocampal gyrus /fusiform gyrus/occipital gyrus/lingual gyrus/cuneus | -- | 7724 | -28 | -25 | -19 | 346.01 | 0.771 |
| L amygdala/parahippocampal gyrus | 28 | 51 | -16 | -7 | -10 | 54.57 | 0.346 |
|  |  |  |  |  |  |  |  |
| ***Emotion*** |  |  |  |  |  |  |  |
|  |  |  |  |  |  |  |  |
| R middle frontal gyrus | 10 | 136 | 32 | 41 | 14 | 30.57 | 0.229 |
| L medial frontal gyrus | 6 | 62 | -22 | 2 | 50 | 30.13 | 0.226 |
| R superior frontal gyrus | 8 | 46 | 2 | 35 | 44 | 19.40 | 0.158 |
| R middle frontal gyrus | 6 | 32 | 29 | 14 | 53 | 20.32 | 0.165 |
| L superior temporal gyrus | 22 | 148 | 62 | -10 | 5 | 36.75 | 0.263 |
| L superior temporal gyrus | 42 | 70 | -61 | -22 | 8 | 33.51 | 0.245 |
| L superior temporal gyrus | 22 | 32 | -58 | -4 | 8 | 26.08 | 0.202 |
| L precentral gyrus | 6 | 37 | -28 | -19 | 53 | 28.14 | 0.214 |
| L/R cuneus/lingual gyrus | 18/17/23 | 736 | 11 | -82 | 14 | 32.10 | 0.238 |
| L inferior occipital gyrus/fusiform gyrus | 18 | 443 | -28 | -85 | -7 | 61.57 | 0.374 |
| R middle occipital gyrus/ fusiform gyrus | 19 | 369 | 35 | -79 | -10 | 54.51 | 0.346 |
| R thalamus | -- | 32 | 11 | -4 | 5 | 39.20 | 0.276 |
| L inferior semi-lunar lobule | -- | 63 | -31 | -70 | -43 | 26.39 | 0.204 |
| R angular gyrus | 40 | 52 | 38 | -55 | 35 | 21.00 | 0.169 |
| L pyramis | -- | 32 | -16 | -79 | -31 | 21.60 | 0.173 |
| L declive | -- | 30 | -16 | -61 | -19 | 22.54 | 0.180 |
|  |  |  |  |  |  |  |  |
| ***Direction-by-Type*** |  |  |  |  |  |  |  |
|  |  |  |  |  |  |  |  |
| L inferior temporal gyrus | 37 | 100 | -49 | -64 | 2 | 30.91 | 0.231 |
| R cuneus | 18 | 30 | 17 | -82 | 26 | 21.50 | 0.172 |
| L/R lingual gyrus/cuneus | 18/17 | 186 | -7 | -74 | 5 | 27.73 | 0.212 |
| R middle occipital gyrus | 19 | 130 | 38 | -70 | 8 | 29.02 | 0.220 |
| R fusiform gyrus | 37 | 45 | 47 | -55 | -10 | 27.06 | 0.208 |
| R fusiform gyrus | 37 | 23 | 26 | -49 | -7 | 22.21 | 0.177 |
|  |  |  |  |  |  |  |  |
| ***Type-by-Emotion*** |  |  |  |  |  |  |  |
|  |  |  |  |  |  |  |  |
| L/R PCC/cuneus | 30/31 | 541 | 8 | -64 | 11 | 28.26 | 0.215 |
| L inferior occipital gyrus | 18 | 156 | -25 | -88 | -10 | 40.86 | 0.284 |
| R lingual gyrus | 18 | 141 | 26 | -91 | -4 | 34.40 | 0.250 |
| R middle occipital gyrus | 37 | 122 | 53 | -67 | 5 | 31.17 | 0.232 |
| L middle occipital gyrus | 19 | 36 | -43 | -76 | 8 | 20.28 | 0.165 |
|  |  |  |  |  |  |  |  |

Key to Table S3: ^a^ According to the Talairach Daemon Atlas (<http://www.nitrc.org/projects/tal-daemon/>), ^b^ Based on the Tournoux & Talairach standard brain template. All results presented at *p* < 0.001 (corrected *p* < 0.05). $\eta_{p}^{2}$ = partial eta squared.

Table S4. Brain regions displaying significant task variable interactions with ARI and/or ICU, obtained from the irritability-by-CU traits-by-Direction-by-Type-by-Emotion repeated measures ANCOVA for the participants who did not take SSRIs.

| **Region^a^** | **BA** | **Voxels** | **X** | **Y** | **Z^b^** | ***F*-value** | $\boldsymbol{\eta}_{\boldsymbol{p}}^{\boldsymbol{2}}$ |
| --- | --- | --- | --- | --- | --- | --- | --- |
|  |  |  |  |  |  |  |  |
| ***Irritability-by-CU traits-by-Direction-by-Emotion*** |  |  |  |  |  |  |  |
|  |  |  |  |  |  |  |  |
| R thalamus/PAG | -- | 22 | 11 | -37 | 5 | 27.39 | 0.239 |
| L lingual gyrus*** | 18 | 74 | -22 | -55 | 2 | 20.13 | 0.188 |
| R culmen/fusiform gyrus | 36 | 24 | 38 | -34 | -25 | 28.08 | 0.244 |
| L ACC*** | 10 | 7 | -13 | 41 | 8 | 14.60 | 0.144 |
| L PCC*** | 31 | 29 | -10 | -52 | 26 | 17.97 | 0.171 |
|  |  |  |  |  |  |  |  |
| ***Irritability-by-Direction-by-Emotion*** |  |  |  |  |  |  |  |
|  |  |  |  |  |  |  |  |
| L ACC | 24 | 34 | -4 | 35 | 5 | 18.32 | 0.174 |
| L PCC | -- | 20 | -13 | -46 | 20 | 25.84 | 0.229 |
|  |  |  |  |  |  |  |  |
| ***CU traits-by-Direction-by-Type*** |  |  |  |  |  |  |  |
|  |  |  |  |  |  |  |  |
| R middle frontal gyrus | 9 | 74 | 35 | 41 | 29 | 27.37 | 0.239 |
| L superior frontal gyrus | 10 | 38 | -25 | 53 | 14 | 21.91 | 0.201 |
| L precuneus | 7 | 23 | -1 | -52 | 59 | 18.48 | 0.175 |
|  |  |  |  |  |  |  |  |

Key to Table S4: ^a^ According to the Talairach Daemon Atlas (<http://www.nitrc.org/projects/tal-daemon/>), ^b^ Based on the Tournoux & Talairach standard brain template. All results presented at *p* < 0.001 (corrected *p* < 0.05) except *** (*p* < 0.005). $\eta_{p}^{2}$ = partial eta squared.

Table S5. Brain regions displaying significant task variable interactions with ARI and/or ICU, obtained from the irritability-by-CU traits-by-Direction-by-Type-by-Emotion repeated measures ANCOVA for the participants who did not take antipsychotic medications.

| **Region^a^** | **BA** | **Voxels** | **X** | **Y** | **Z^b^** | ***F*-value** | $\boldsymbol{\eta}_{\boldsymbol{p}}^{\boldsymbol{2}}$ |
| --- | --- | --- | --- | --- | --- | --- | --- |
|  |  |  |  |  |  |  |  |
| ***Irritability-by-CU traits-by-Direction-by-Emotion*** |  |  |  |  |  |  |  |
|  |  |  |  |  |  |  |  |
| R thalamus/PAG | -- | 28 | 11 | -37 | 5 | 32.51 | 0.270 |
| L lingual gyrus*** | 30 | 48 | -19 | -61 | 5 | 17.69 | 0.174 |
| L ACC*** | 10 | 39 | -13 | 41 | 11 | 11.29 | 0.118 |
| L PCC*** | 31 | 20 | -10 | -52 | 26 | 12.34 | 0.128 |
|  |  |  |  |  |  |  |  |
| ***Irritability-by-Direction-by-Emotion*** |  |  |  |  |  |  |  |
|  |  |  |  |  |  |  |  |
| L ACC | 24 | 38 | -4 | 35 | 5 | 18.44 | 0.173 |
| L PCC | -- | 23 | -13 | -46 | 23 | 26.52 | 0.232 |
| L fusiform gyrus | -- | 23 | -25 | -28 | -13 | 25.68 | 0.226 |
|  |  |  |  |  |  |  |  |
| ***Irritability-by-Direction*** |  |  |  |  |  |  |  |
|  |  |  |  |  |  |  |  |
| L cerebellar lingual | -- | 23 | -1 | -46 | -13 | 22.85 | 0.206 |
|  |  |  |  |  |  |  |  |

Key to Table S5: ^a^ According to the Talairach Daemon Atlas (<http://www.nitrc.org/projects/tal-daemon/>), ^b^ Based on the Tournoux & Talairach standard brain template. All results presented at *p* < 0.001 (corrected *p* < 0.05) except *** (*p* < 0.005). $\eta_{p}^{2}$ = partial eta squared.

Table S6. Brain regions displaying significant task variable interactions with ARI and/or ICU, obtained from the irritability-by-CU traits-by-Direction-by-Type-by-Emotion repeated measures ANCOVA for the participants who did not take stimulants.

| **Region^a^** | **BA** | **Voxels** | **X** | **Y** | **Z^b^** | ***F*-value** | $\boldsymbol{\eta}_{\boldsymbol{p}}^{\boldsymbol{2}}$ |
| --- | --- | --- | --- | --- | --- | --- | --- |
|  |  |  |  |  |  |  |  |
| ***Irritability-by-CU traits-by-Direction-by-Emotion*** |  |  |  |  |  |  |  |
|  |  |  |  |  |  |  |  |
| R thalamus/PAG*** | 27/30 | 61 | 11 | -37 | 5 | 27.40 | 0.248 |
| L lingual gyrus | 18 | 28 | -22 | -55 | 2 | 20.99 | 0.202 |
| R culmen/fusiform gyrus | -- | 31 | 38 | -37 | -25 | 27.07 | 0.246 |
| L ACC*** | 60 | 32 | -10 | 35 | 17 | 15.93 | 0.161 |
| L PCC*** | 31 | 21 | -10 | -52 | 26 | 15.59 | 0.158 |
|  |  |  |  |  |  |  |  |
| ***Irritability-by-Direction-by-Emotion*** |  |  |  |  |  |  |  |
|  |  |  |  |  |  |  |  |
| L ACC*** | 24 | 38 | -4 | 35 | 5 | 13.88 | 0.143 |
| L PCC*** | 31 | 92 | -13 | -46 | 23 | 18.46 | 0.182 |
| R amygdala/parahippocampal gyrus*** | 28 | 51 | 20 | -16 | -19 | 25.57 | 0.236 |
| L fusiform gyrus*** | 28 | 41 | -25 | -28 | -13 | 22.91 | 0.216 |
|  |  |  |  |  |  |  |  |
|  |  |  |  |  |  |  |  |
| ***Irritability-by-Type*** |  |  |  |  |  |  |  |
|  |  |  |  |  |  |  |  |
| R superior temporal gyrus | 41 | 25 | 38 | -31 | 8 | 43.62 | 0.345 |
|  |  |  |  |  |  |  |  |

Key to Table S6: ^a^ According to the Talairach Daemon Atlas (<http://www.nitrc.org/projects/tal-daemon/>), ^b^ Based on the Tournoux & Talairach standard brain template. All results presented at *p* < 0.001 (corrected *p* < 0.05) except *** (*p* < 0.005). $\eta_{p}^{2}$ = partial eta squared.

Table S7. Brain regions displaying significant task variable interactions with ARI, obtained from the 2 (Direction: Looming, Receding) by 2 (Type: Human, Animal) by 2 (Emotion: Threatening, Neutral) ANCOVA with ARI as a single continuous covariate for all the participants.

| **Region^a^** | **BA** | **Voxels** | **X** | **Y** | **Z^b^** | ***F*-value** | $\boldsymbol{\eta}_{\boldsymbol{p}}^{\boldsymbol{2}}$ |
| --- | --- | --- | --- | --- | --- | --- | --- |
|  |  |  |  |  |  |  |  |
| ***Irritability-by-Direction-by-Emotion*** |  |  |  |  |  |  |  |
|  |  |  |  |  |  |  |  |
| L ACC | 24 | 47 | -4 | 35 | 5 | 17.88 | 0.148 |
| R amygdala/parahippocampal gyrus | -- | 26 | 20 | -10 | -16 | 27.43 | 0.210 |
|  |  |  |  |  |  |  |  |

Key to Table S7: ^a^ According to the Talairach Daemon Atlas (<http://www.nitrc.org/projects/tal-aemon/>), ^b^ Based on the Tournoux & Talairach standard brain template. All results presented at *p* < 0.001 (corrected *p* < 0.05). $\eta_{p}^{2}$ = partial eta squared.

Table S8. Brain regions displaying significant task variable interactions with ICU, obtained from the 2 (Direction: Looming, Receding) by 2 (Type: Human, Animal) by 2 (Emotion: Threatening, Neutral) ANCOVA with ICU as a single continuous covariate for all the participants.

| **Region^a^** | **BA** | **Voxels** | **X** | **Y** | **Z^b^** | ***F*-value** | $\boldsymbol{\eta}_{\boldsymbol{p}}^{\boldsymbol{2}}$ |
| --- | --- | --- | --- | --- | --- | --- | --- |
|  |  |  |  |  |  |  |  |
| ***CU traits-by-Direction-by-Type*** |  |  |  |  |  |  |  |
|  |  |  |  |  |  |  |  |
| R middle frontal gyrus | 9 | 76 | 35 | 41 | 29 | 27.37 | 0.218 |
| L middle frontal gyrus | 9 | 31 | -28 | 32 | 32 | 22.78 | 0.189 |
| L superior frontal gyrus | -- | 29 | -25 | 44 | 14 | 19.78 | 0.168 |
|  |  |  |  |  |  |  |  |

Key to Table S8: ^a^ According to the Talairach Daemon Atlas (<http://www.nitrc.org/projects/tal-aemon/>), ^b^ Based on the Tournoux & Talairach standard brain template. All results presented at *p* < 0.001 (corrected *p* < 0.05). $\eta_{p}^{2}$ = partial eta squared.

Table S9. Brain regions displaying significant irritability-by-CU traits-by-Direction-by-Emotion interactions and irritability-by-Direction-by-Emotion interactions but no effects associated with age, obtained from the irritability-by-CU traits-by-age-by-Direction-by-Type-by-Emotion repeated measures ANCOVA for all the participants.

| **Region^a^** | **BA** | **Voxels** | **X** | **Y** | **Z^b^** | ***F*-value** | $\boldsymbol{\eta}_{\boldsymbol{p}}^{\boldsymbol{2}}$ |
| --- | --- | --- | --- | --- | --- | --- | --- |
|  |  |  |  |  |  |  |  |
| ***Irritability-by-CU traits-by-Direction-by-Emotion*** |  |  |  |  |  |  |  |
|  |  |  |  |  |  |  |  |
| R thalamus/PAG | 27/30 | 33 | 11 | -37 | 5 | 35.39 | 0.269 |
| L lingual gyrus*** | 30 | 82 | -19 | -58 | 5 | 23.08 | 0.194 |
| R culmen/fusiform gyrus*** | -- | 38 | 38 | -34 | -25 | 27.39 | 0.222 |
| L ACC extended to dmPFC*** | 8 | 205 | -7 | 47 | 44 | 17.63 | 0.155 |
| L PCC*** | 31 | 35 | -10 | -52 | 26 | 16.66 | 0.148 |
|  |  |  |  |  |  |  |  |
| ***Irritability-by-Direction-by-Emotion*** |  |  |  |  |  |  |  |
|  |  |  |  |  |  |  |  |
| L ACC*** | 24 | 71 | -4 | 35 | 5 | 15.76 | 0.141 |
| L PCC*** | -- | 99 | -13 | -46 | 23 | 19.81 | 0.171 |
| R amygdala/parahippocampal gyrus*** | 28 | 51 | 20 | -16 | -19 | 26.36 | 0.215 |
|  |  |  |  |  |  |  |  |

Key to Table S9: ^a^ According to the Talairach Daemon Atlas (<http://www.nitrc.org/projects/tal-daemon/>), ^b^ Based on the Tournoux & Talairach standard brain template. All results presented at *p* < 0.001 (corrected *p* < 0.05) except *** (*p* < 0.005). $\eta_{p}^{2}$ = partial eta squared.

Table S10. Significant areas of activation obtained from the age-by-Direction-by-Type-by-Emotion repeated measures ANCOVA for the independent TD sample.

| **Region^a^** | **BA** | **Voxels** | **X** | **Y** | **Z^b^** | ***F*-value** | ***ηp*²** |
| --- | --- | --- | --- | --- | --- | --- | --- |
|  |  |  |  |  |  |  |  |
| ***Age-by-Direction-by-Type-Emotion*** |  |  |  |  |  |  |  |
|  |  |  |  |  |  |  |  |
| L/R rmPFC | 10 | 65 | -1 | 50 | 2 | 24.68 | 0.203 |
| L/R dmPFC | 9 | 131 | 5 | 41 | 23 | 21.10 | 0.179 |
| L superior frontal gyrus | 8 | 94 | -19 | 32 | 50 | 23.72 | 0.196 |
| L cingulate gyrus | 31 | 137 | -1 | -43 | 29 | 24.47 | 0.201 |
| L middle temporal gyrus | 39 | 41 | -52 | -64 | 20 | 25.15 | 0.206 |
| L cerebellar tonsil | -- | 26 | -22 | -64 | -34 | 27.41 | 0.220 |
|  |  |  |  |  |  |  |  |
| ***Direction*** |  |  |  |  |  |  |  |
|  |  |  |  |  |  |  |  |
| *Looming > Receding* |  |  |  |  |  |  |  |
| L/R fusiform gyrus/occipital gyrus/cuneus/lingual gyrus | -- | 4264 | -28 | -64 | -13 | 168.56 | 0.635 |
| L thalamus | -- | 62 | -19 | -22 | -1 | 68.01 | 0.412 |
| R thalamus | -- | 42 | 20 | -25 | 2 | 43.09 | 0.308 |
| R amygdala/parahippocampal gyrus | 28 | 23 | -19 | -4 | -13 | 25.55 | 0.208 |
| R amygdala/parahippocampal gyrus | 28 | 86 | 20 | -4 | -13 | 44.57 | 0.315 |
|  |  |  |  |  |  |  |  |
| *Looming < Receding* |  |  |  |  |  |  |  |
| R middle frontal gyrus | 9 | 32 | 35 | 29 | 35 | 21.84 | 0.184 |
| R middle temporal gyrus | -- | 43 | 38 | -58 | 5 | 30.93 | 0.242 |
| R inferior parietal lobule | 40 | 55 | 44 | -43 | 50 | 23.92 | 0.198 |
| R inferior occipital gyrus | 18 | 37 | 26 | -91 | -7 | 50.50 | 0.342 |
| ***Type*** |  |  |  |  |  |  |  |
|  |  |  |  |  |  |  |  |
| *Human > Animal* |  |  |  |  |  |  |  |
| L/R vmPFC | 10 | 186 | 5 | 44 | -7 | 43.99 | 0.312 |
| R rmPFC | 9 | 45 | 5 | 56 | 26 | 25.92 | 0.211 |
| L/R ACC | 25 | 38 | 5 | 8 | -7 | 37.29 | 0.278 |
| R superior temporal gyrus | -- | 201 | 47 | -40 | 11 | 38.04 | 0.282 |
| R middle temporal gyrus | -- | 61 | 53 | -61 | 29 | 32.38 | 0.250 |
| R superior temporal gyrus | 22 | 38 | 47 | -13 | -7 | 25.21 | 0.206 |
| L middle temporal gyrus | -- | 29 | -49 | -10 | -10 | 18.21 | 0.158 |
| L inferior parietal lobule | 7 | 68 | -43 | -64 | 44 | 21.42 | 0.181 |
| L/R precuneus | 31 | 274 | 5 | -49 | 35 | 32.40 | 0.250 |
| R amygdala/parahippocampal gyrus | -- | 23 | 20 | -7 | -10 | 31.92 | 0.248 |
| *Human < Animal* |  |  |  |  |  |  |  |
| L/R parahippocampal gyrus /fusiform gyrus/occipital gyrus/lingual gyrus/cuneus | -- | 7852 | -31 | -22 | -19 | 403.00 | 0.806 |
| L cerebellum tonsil | -- | 23 | -13 | -37 | -43 | 29.58 | 0.234 |
|  |  |  |  |  |  |  |  |
| ***Emotion*** |  |  |  |  |  |  |  |
|  |  |  |  |  |  |  |  |
| *Threatening > Neutral* |  |  |  |  |  |  |  |
| R middle occipital gyrus | 18 | 559 | 29 | -85 | -1 | 88.78 | 0.478 |
| L middle occipital gyrus/fusiform gyrus | 18 | 554 | -34 | -82 | -10 | 71.29 | 0.424 |
| L subcallosal gyrus | 34 | 27 | -16 | 8 | -13 | 29.05 | 0.230 |
|  |  |  |  |  |  |  |  |
| *Threatening < Neutral* |  |  |  |  |  |  |  |
| R cuneus | 18 | 64 | 8 | -82 | 17 | 22.73 | 0.190 |
|  |  |  |  |  |  |  |  |
| ***Direction-by-Type*** |  |  |  |  |  |  |  |
|  |  |  |  |  |  |  |  |
| L inferior temporal gyrus | 37 | 69 | -46 | -61 | -1 | 37.27 | 0.278 |
| R fusiform gyrus | 19 | 62 | 29 | -52 | -7 | 34.05 | 0.260 |
| L lingual gyrus/cuneus | 19 | 314 | -16 | -64 | -4 | 30.21 | 0.237 |
| R lingual gyrus | 18 | 124 | 8 | -76 | 5 | 27.32 | 0.220 |
| L parahippocampal gyrus | 37 | 46 | -28 | -43 | -10 | 28.16 | 0.225 |
|  |  |  |  |  |  |  |  |
| ***Type-by-Emotion*** |  |  |  |  |  |  |  |
|  |  |  |  |  |  |  |  |
| L cuneus | 17 | 95 | -10 | -76 | 11 | 20.11 | 0.172 |
| R cuneus | 31 | 30 | 20 | -67 | 8 | 18.84 | 0.163 |
| R inferior occipital gyrus | 18 | 236 | 29 | -85 | -7 | 44.46 | 0.314 |
| L lingual gyrus | 17/18 | 163 | -22 | -91 | -4 | 47.63 | 0.329 |
| R culmen | 18 | 43 | 11 | -64 | -4 | 21.08 | 0.179 |
|  |  |  |  |  |  |  |  |

Key to Table S10: ^a^ According to the Talairach Daemon Atlas (<http://www.nitrc.org/projects/tal-daemon/>), ^b^ Based on the Tournoux & Talairach standard brain template. All results presented at *p* < 0.001 (corrected *p* < 0.05) except *** (*p* < 0.005). $\eta_{p}^{2}$ = partial eta squared.

**
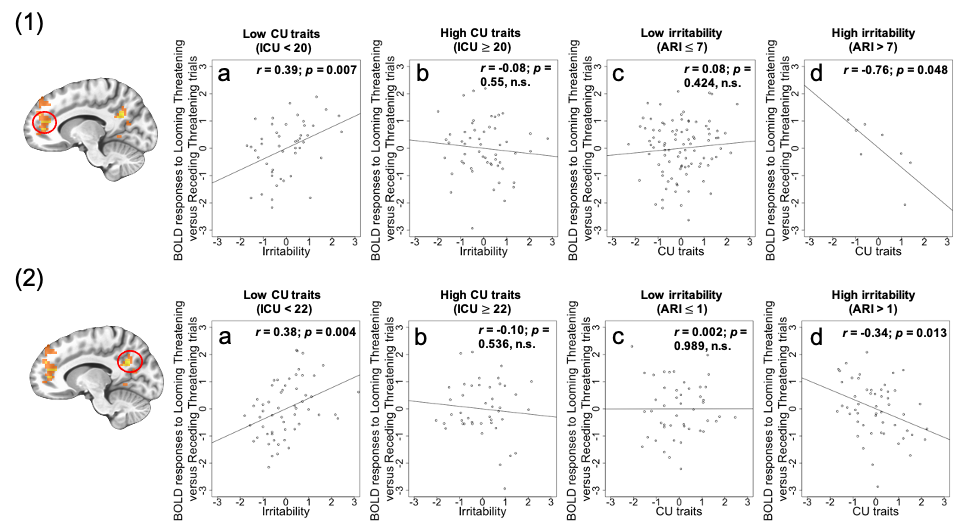
**

Figure S1. (1) Ventral ACC (coordinates: -13, 41, 8) showing a significant irritability-by-CU traits-by-Direction-by-Emotion interaction; (2) PCC (coordinates: -10, -52, 26) showing a significant irritability-by-CU traits-by-Direction-by-Emotion interaction. Scatterplots depict the partial correlations within these regions: Adjusted residuals for the BLOM transformed z-scored ARI scores or ICU scores (x-axis) are plotted against adjusted residuals for the average differential BOLD responses to Looming Threatening versus Receding Threatening trials (y-axis). *r*s are all partial. 1a and 2a: ARI score was significantly positively associated with the Looming Threatening versus Receding Threatening differential response for participants with lower ICU scores; 1b and 2b: This association was not significant for participants with higher ICU scores; 1c and 2c: ICU score was not significantly correlated with the Looming Threatening versus Receding Threatening differential response for participants with lower ARI scores; 1d and 2d: This association was significant for participants with higher ARI score.
